# The effect of antimicrobial drug use on the composition of the genitourinary microbiota in an elderly population

**DOI:** 10.1186/s12866-018-1379-1

**Published:** 2019-01-09

**Authors:** M. Mulder, D. Radjabzadeh, R. J. Hassing, J. Heeringa, A. G. Uitterlinden, R. Kraaij, B. H. Stricker, A. Verbon

**Affiliations:** 1 Department of Epidemiology, Erasmus Medical Center, PO Box 2040, 3000 CA Rotterdam, The Netherlands; 2 Inspectorate of Health Care, PO Box 2518, 6401 DA Heerlen, The Netherlands; 3 Department of Internal Medicine, Erasmus Medical Center, PO Box 2040, 3000 CA Rotterdam, The Netherlands; 4 Department of Internal Medicine, Rijnstate Hospital, PO Box 9555, 6800 TA Arnhem, The Netherlands; 5 Department of Medical Microbiology and Infectious Diseases, Erasmus Medical Center, PO Box 2040, 3000 CA Rotterdam, The Netherlands

**Keywords:** Genitourinary microbiota, Antimicrobial drug use, Urinary tract infections

## Abstract

**Background:**

The urinary tract is inhabited by a diversity of microorganisms, known as the genitourinary microbiota. Here, we investigated the association between the use of antimicrobial drugs and the composition of the genitourinary microbiota.

**Results:**

Clean-catch urinary samples were collected from 27 participants of the Rotterdam Study. Bacterial DNA was extracted and the 16S ribosomal RNA gene variable regions V3 and V4 were analyzed using Illumina sequencing. 23 of the 27 participants were included in the analysis. The population consisted of 10 men and 13 women with a mean age of 75 ± 3 years. The time between the last prescription of an antimicrobial drug and sampling was determined and categorized. The use of antimicrobial drugs prior to urine sampling was associated with statistically significant differences in the beta-diversity of the genitourinary microbiota. No association was found between antimicrobial drug use and the alpha-diversity of the genitourinary microbiota. Operational Taxonomic Units (OTUs) that were lowest in participants who used antimicrobial drug belonged to *Lactobacillus* and *Finegoldia.* In contrast, an OTU belonging to the genus *Parabacteroides* had higher abundances. Also, an OTU belonging to the species *E*.*coli* was higher in the participants who used antimicrobial drugs.

**Conclusion:**

Prior use of antimicrobial drugs is associated with a different composition of the genitourinary microbiota. Our results might indicate a persisting effect of antimicrobial drugs on the composition of the microbiota, but reverse causality cannot be ruled out. Future studies are needed to differentiate between two possibilities. Genitourinary dysbiosis could be the result of antimicrobial drug use or genitourinary dysbiosis could be a risk factor for urinary tract infections resulting in increased use of antimicrobial drugs. This may have important implications for treatment and prevention of (recurrent) UTIs.

## Background

The term microbiota, which is often interchangeably used with the microbiome, is defined as the microorganisms that live in a particular body compartment [1]. The microbiota of the gut are the most well-known microbiota and have been described in many studies. Currently, we know that several other body compartments, such as the skin, nose and urinary tract, also have a distinct microbiota and it is assumed that these microbiota are associated with overall health [2].

For long, it was thought that urine was sterile, and the presence of microorganisms in the urinary tract was considered to occur only as part of an infection. In 1979, it was recognized that slow-growing micro-organisms were missed when standard culturing techniques were used [3]. However, it was only with the development of 16S ribosomal RNA sequencing that it was established that most body sites are colonized with bacteria, but the urinary tract was not tested in the Human Microbiome Project [4]. Recently, the microbiota unique to the urinary tract have been reported both in males and females [5, 6]. In females, the microbiota seem to be more complex with higher interindividual variability than in males [7], but no clear relation with urinary tract infections (UTIs) has been demonstrated until now. No evident core microbiota have been found yet, however, this could possibly be present when grouping by age [7, 8].

With the discovery of the urinary microbiota, the interest is growing. Until now, most studies have included small numbers of individuals and have shown considerable variation in the (genito)urinary microbiota within the study population. Nevertheless, several studies have already suggested a dysbiosis of the (genito) urinary microbiota in diseases such as urgency urinary incontinence [9–12].

Another factor that might influence the genitourinary tract microbiota is the use of antimicrobial drugs. For the gut microbiota, it has already been shown that use of antimicrobial drugs (temporarily) influences the composition of the microbiota [13–15]. This effect has not yet been demonstrated for the genitourinary microbiota, despite the fact that antibiotic drugs are very often prescribed for urinary tract infections and have a good penetration in the urinary tract. Here, we investigated the association between the use of antimicrobial drugs and the composition of the genitourinary microbiota.

## Results

Sufficient bacterial DNA could be obtained in 24 of the 27 participants (88.9%), whereas 3 participants (two males, one female) were excluded from the analysis because the DNA obtained was not sufficient for the analyses. Additionally, 1 participant was excluded because the composition of her microbiota consisted for 99.9% of *Escherichia coli.* Unfortunately, we do not know whether she had symptoms indicating a UTI, thus we could not exclude a UTI at the time of sampling. Therefore, the study population consisted of 10 (45.5%) males and 13 (56.5%) females with a median age of 75 years (range 71–83 years). Of all participants, 7 (30.4%) had used antimicrobial drugs in the previous year (Table 1). The microbiota compositions showed considerable variability between participants. The most abundantly detected phyla were Firmicutes, Bacteroidetes and Proteobacteria, in descending order, whereas the most abundant species was *Escherichia coli*. We did not find any differences in alpha-diversity and beta-diversity between men and women.

**Table 1:** Basic characteristics of the study population

Characteristic	Value
Age, median (IQR)	74.7 (73.1–77.0)
Sex (female), *n* (%)	13 (56.5)
Diabetes	4 (17.4)
Kidney function	84.4 (74.5–94.6)
Antimicrobial drug use, *n* (%)	
No use	2 (8.7)
> 96 months	4 (17.4)
73–96 months	1 (4.3)
49–72 months	1 (4.3)
25–48 months	3 (13.0)
13–24 months	5 (21.7)
0–12 months	7 (30.4)

Table 1 shows the basic characteristics of the study population. Diabetes was assessed as the use of antidiabetic medication. The kidney function indicated the glomerular filtration rate, which was calculated with the *CKD-EPI equation.* The use of any antimicrobial drug (except for J01F) before sampling was categorized in 0–12 months (6), 13–24 months (5), 25–48 months (4), 49–72 months (3), 73–96 months (2) and > 96 months (1) before sampling or no use (0)

**Fig. 1:**
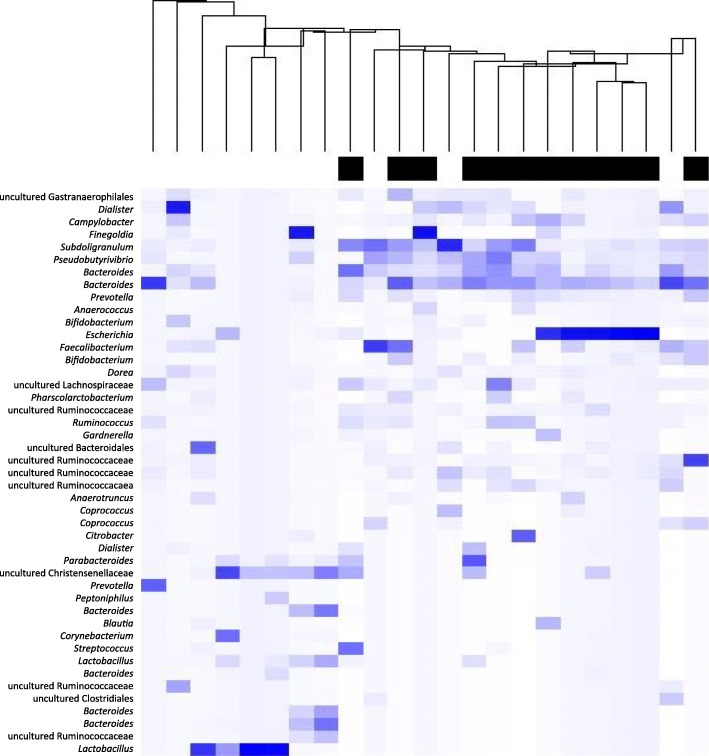
Heatmap of urinary microbiota. Heatmap of bacterial profiles of individuals who used antimicrobial drugs in 48 months before (black) and individuals who used no antimicrobial drugs or longer than 48 months before sampling (white). These analyses were performed on a cleaned dataset, showing the most abundant OTUs. Bray-Curtis dissimilarities were used to determine differences and median linkage was used for hierarchical clustering of samples

Also, no difference could be demonstrated in alpha-diversities for antimicrobial drug use, indicating that the diversity of the microbiota was not different in the participants that had used antimicrobial drug use. However, the beta-diversity after (categorized) antimicrobial drug use was significantly different (*p* < 0.005), meaning that the use of antimicrobial drugs is clearly associated with a different composition of the microbiota. This difference was still present after adjustment for sex, age, diabetes and kidney function (*p* < 0.005) (Fig. 1 and Table 2). Since other antimicrobial drug prescriptions prior to the last prescription before sampling could also have influenced the genitourinary microbiota, a confounder that represented the number of other antimicrobial drug prescriptions since approximately 1995 was added in a sensitivity analysis. This did not influence the results (*p* < 0.005). In another sensitivity analysis where macrolides were added to antimicrobial drug use, the community structure was also different after antimicrobial drug use (*p* = 0.03) and borderline significant after adjustment for confounders (*p* = 0.05).

**Table 2:** Genera that differ after antimicrobial drug use

Lower after antimicrobial drug use		Higher after antimicrobial drug use	
Genus	Estimate	Genus	Estimate
*Lactobacillus*	−0.054	*Parabacteroides*	0.046
*Finegoldia*	−0.054	uncultured Ruminococcaceae	0.041
uncultured FamilyXI	−0.053	*Bacteroides*	0.039
*Helcococcus*	−0.053	uncultured Defluviitaleaceae	0.037
*Gallicola*	−0.052	*Escherichia*	0.036
*Helcococcus*	−0.051	*Faecalibacterium*	0.036
*Streptococcus*	−0.051	uncultured Ruminococcaceae	0.035
*Streptococcus*	−0.050	*Intestinimonas*	0.032
*Porphyromonas*	−0.049	*Anaerotruncus*	0.031
*Porphyromonas*	−0.049	*Bacteroides*	0.029
*Facklamia*	−0.047	*Blautia*	0.029
*Dialister*	−0.043	*Barnesiella*	0.029
*Alloscardovia*	−0.043	*Bacteroides*	0.028
*Anaerococcus*	−0.041	*Blautia*	0.027
*Prevotella*	−0.040	*Pseudobutyrivibrio*	0.027
*Peptoniphilus*	−0.039	*Bacteroides*	0.025
*Dialister*	−0.033	uncultured Lachnospiraceae	0.025
*Howardella*	−0.031	uncultured Ruminococcaceae	0.025
*Roseburia*	−0.022	uncultured Defluviitaleaceae	0.024
*Porphyromonas*	−0.015	*Bifidobacterium*	0.024
*Prevotella*	−0.014	*uncultured* Ruminococcaceae	0.022
*Actinotignum* (formerly known as *Actinobaculum*)	−0.012	*uncultured* Lachnospiraceae	0.022
*Actinotignum* (formerly known as *Actinobaculum*)	−0.006	*Bacteroides*	0.013
*Fusobacterium*	−0.004		

Table 2 shows differences in genera estimated with the MaAsLin analysis. It shows genera that corresponds with OTUs that significantly differed (*p* < 0.05) after antimicrobial drug use. Antimicrobial drug use was analyzed as follows: *no use (0), use > 96 months before sampling (1), use 73–96 months before sampling (2), use 49–72 months before sampling (3), use 25–48 months before sampling (4), use 13–24 months before sampling (5), use 0–12 months before sampling (6). The estimate is a measure of the strength of the association where negative estimates mean that the OTU is lower in users of antimicrobial drugs, whereas the positive OTUs are higher in users of antimicrobial drugs. Duplicate genera refer to different OTUs of the same genus*

Several OTUs were shown to be significantly lower or higher in participants who used antimicrobial drugs than in those who did not. The OTUs that were most reduced in participants who had used antimicrobial drugs belonged to the genera *Lactobacillus* and a *Finegoldia,* followed by an uncultured member of FamilyXI, 2 OTUs belonging to the genus *Helcococcus*, an OTU belonging to the genus *Gallicola*, 2 OTUs belonging to the genus *Streptococcus and* 2 OTUs belonging to the genus *Porphyromonas*. Of the OTUs that were higher after antimicrobial drug use, one of the strongest belonged to the genus *Escherichia*. Other OTUs that were higher included members of the genera *Parabacteroides*, *Bacteroides* and *Faecalibacterium* as well as uncultured members of the families Ruminococcaceae and Defluviitaleaceae (Table 2).

## Discussion

In this study of elderly asymptomatic persons, we showed that previous use of antimicrobial drugs is associated with differences in the composition of the genitourinary microbiota. OTUs that were lowest in participants who used antimicrobial drugs belonged to the genera *Lactobacillus* and a *Finegoldia.* In contrast, an OTU belonging to the species *E.coli* was higher in participants who used antimicrobial drugs.

We here showed a difference in the genitourinary microbiota after the use of antimicrobial drugs. We only considered the last prescription before urinary sampling, which most likely has had the strongest influence on the genitourinary microbiota. However, it could be assumed that other prior prescriptions could also have influenced the genitourinary microbiota, but additional adjustment for the number of prior drug prescriptions did not influence the results. Due to this and due to the cross-sectional study design, our results do not differentiate between antimicrobial treatment as a cause for dysbiosis versus the possibility that long-term dysbiosis was the cause of UTIs and subsequent antimicrobial treatment. This must be considered, since it has been hypothesized that UTIs are the result of dysbiosis of the microbiota in the genitourinary tract [16]. There are several arguments in favor of the hypothesis that dysbiosis of the genitourinary microbiota has caused UTIs, resulting in antimicrobial treatment. First, several of the antimicrobial drugs groups that have been prescribed to our participants, e.g. sulfonamides and trimethoprim (J01E) and nitrofuran derivatives (J01XE) are mainly, if not solely, prescribed for urinary tract infections by general practitioners (GPs) in the Netherlands. Second, in our population the dysbiosis was persistent for years after stopping the antimicrobial drugs. Although, this could also mean that antimicrobial drugs can have a persistent effect on the genitourinary microbiota.

A few other studies investigated the effects of antimicrobial drugs on the genitourinary microbiota or the effect of a specific composition of the genitourinary microbiota on UTIs. Differences were demonstrated in the urinary microbiota of kidney transplant patients who received prophylactic trimethoprim-sulfamethoxazole treatment compared to healthy controls, indicating that the genitourinary microbiota may be modified by antimicrobial drugs use [17]. In contrast to our study, the genitourinary microbiota of patients using trimethoprim-sulfamethoxazole had a decreased microbial diversity compared to healthy controls. This may be due to current versus past antimicrobial drug use, age and use of immunosuppressive medication of the kidney transplant patients, and the fact that this group differs from community-dwelling elderly [17]. Another study has already shown associations between the urinary microbiota and UTIs. Differences were shown in the microbiota of women on the day of surgery between women who did or did not develop a post-operative UTI [18]. However, one might also argue that changes in microbiota caused by antimicrobial drugs increase susceptibility to UTIs. For instance, it was shown in a cohort with 113 women that 27% experienced at least one recurrence within 6 months after an initial UTI, whereas in a cohort of 179 Finnish women 44% had recurrences [19, 20]. Also, it was shown in mice that transient exposure to *Gardnerella vaginalis*, a member of the vaginal microbiota, can trigger *E.coli* reservoirs in the bladder to cause a UTI [21], which might be an effect of antibiotic use. In our population, *Lactobacillus*, which is thought to play a role in the prevention of UTIs in women [22] was lower in the participants who used antimicrobial drugs. Also, a depletion of *Lactobacillus iners* in urine has recently been associated with postoperative UTI risk. This study also showed that enrichment of a diverse mixture of uropathogens was associated with postoperative UTI [23]. We found that *E.coli* was higher in the participants who had used antimicrobial drugs. It is not clear what the cause is or the consequence, and therefore further studies to elucidate the causal relationship between the genitourinary microbiota and the use of antimicrobial drugs are needed.

The strength of our study is that community-dwelling participants from The Rotterdam Study were included. The Rotterdam Study has prospectively gathered records without prior knowledge of research hypotheses. This includes data on drug prescriptions obtained from a collaborative database of all community pharmacies in the Ommoord area. Furthermore, the performed analyses compared the total microbiota compositions instead of comparing individual elements separately. However, our study also has some limitations. First of all, we only had a small sample size, but even in these small groups we could detect significant differences in microbiota. A second possible limitation may be that all participants were 70 years or older, whereas it has been shown that the diversity of gut microbiota declines after the age of 70 [24], and it has been assumed that the urinary microbiota also change with age [7, 25]. Although, the genera found in the genitourinary microbiota in our study were also found by others, our findings should be extrapolated with care to younger individuals, especially premenopausal women [7]. A third limitation is the methods that we used. We collected midstream urine samples compared to urinary catheterization used in some other studies. Unfortunately, the latter is difficult to accomplish in a community-dwelling cohort of healthy elderly. The participants obtained clear instructions for collecting clean-catch midstream urine and diverse collection methods have indicated that the urinary microbiota is not simply the consequence of contamination or urethral colonization [7, 17]. However, it should be kept in mind that it has been shown that the microbiota from voided urine contains a mixture of urinary and genital tract bacteria and therefore we called it the genitourinary microbiota [26]. Additionally, we used centrifugation to precipitate the bacteria to obtain enough DNA for analysis, but this could have introduced bias, since centrifugation will enrich for bacteria that pellet well. In this study, we excluded three participants from the analyses, due to too little bacterial DNA being present in their sample. This has also occurred in another study (3 out of 16) [7], indicating that it is not always possible to obtain sufficient DNA from urine with the present techniques.

## Conclusions

In conclusion, we have shown that the composition of the genitourinary microbiota is associated with the use of antimicrobial drugs. It is not clear whether genitourinary dysbiosis predisposes for UTI with subsequent antibiotic treatment or that the antibiotic use causes the dysbiosis. Further studies are needed to elucidate the causal relationship between the composition of the genitourinary microbiota, UTIs and the use of antimicrobial drugs.

## Methods

### Source population

Twenty-seven participants were randomly selected from the Rotterdam Study, a prospective population-based cohort study of middle-aged and elderly people in the Ommoord area of Rotterdam [27]*,* were asked to provide a urine sample (November/December 2015). The Rotterdam Study has been approved by the Medical Ethics Committee of the Erasmus MC and by the Ministry of Health, Welfare and Sport of the Netherlands, on the basis of the Wet Bevolkingsonderzoek ERGO. All participants provided written informed consent.

### Microbiome analysis

Samples of first-morning clean-catch midstream urine (~ 50 mL) were collected and centrifuged at 6000 g for 10 min. Supernatants were removed and pellets were resuspended in the remaining urine and stored at − 80 °C. Automated DNA-isolation (Arrow DNA; DiaSorin S.p.A., Saluggia, Italy) was performed using the Arrow DNA kit according to the manufacturer’s instructions and included bead-beating in Lysing Matrix B tubes containing 0.1 mm silica beads (MP Biomedicals, LLC, Bio Connect Life Sciences, Huissen, The Netherlands) using the MagNA Lyser instrument (Roche Diagnostics, Almere, The Netherlands) at 7000 rpm for 45 s. Bacterial 16S rRNA variable regions V3 and V4 were amplified and sequenced using the Illumina MiSeq 2 × 300 base pairs protocol [28]. Phylogenetic multi-sample profiling was performed using an in-house developed pipeline based on the QIIME 1.9.0 and USEARCH version 8.1 software packages [29, 30]. After rarefaction at 10,000 reads per sample, taxonomy was assigned both at genus and species level using the naïve Bayesian RDP classifier [31] and the SILVA database (v119) [32]. Cluster analysis and *MaAsLin* analysis were performed on the dataset at genus level. For heatmap analysis, the Operational Taxonomic Unit (OTU) table was cleaned; singletons and OTUs with minimum count fraction of 0.005% [33] (50 reads) were discarded. In addition, the 4% of genera with the lowest abundance were removed.

### Antimicrobial drug use

The date of the last prescription and the total number of prescriptions before sampling of several antimicrobial drug groups was obtained from a collaborative database of all community pharmacies in the Ommoord area. This included: tetracyclines (J01A), beta-lactams (J01C), sulphonamides and trimethoprim (J01E), macrolides (J01FA), fluoroquinolones (J01MA), nitrofuran derivatives (J01XE) and fosfomycin (J01XX01). Although the proportion of renal excretion differs, all of these antimicrobial drugs have a substantial excretion via urine. Macrolides (J01FA) were only analyzed in a sensitivity analysis because they are mainly excreted by the gallbladder. Cephalosporins and aminoglycosides were not prescribed. The time between the last prescription of one of these drugs and urinary sampling was calculated and categorized into no use (0), use > 96 months before sampling (1), use 73–96 months before sampling (2), use 49–72 months before sampling (3), use 25–48 months before sampling (4), use 13–24 months before sampling (5), use 0–12 months before sampling (6).

### Analysis and statistical methods

Statistical analyses were performed in R [34]. Shannon alpha-diversities (measure of diversity of species within a sample) and Bray-Curtis beta-diversities (measure of diversity of species composition between samples) were calculated. Differences between users of antimicrobial drugs in alpha-diversities were tested using a linear regression analysis with the Shannon alpha-diversity as the response variable and the time category of last antimicrobial drug use as the explanatory variable. Differences in beta-diversities were tested using the MiRKAT package, in which it is possible to test the association between a microbiome community and a phenotype with the aid of semi-parametric kernel machine regression [35]. The analyses on the alpha-diversity and beta-diversity were adjusted for age, sex, diabetes (use of anti-diabetic medication) and kidney function (glomerular filtration rate (GFR) according to the CKD-EPI equation) [36]. In a sensitivity analysis, an additional confounder was included which gave the number of prescriptions of all antimicrobial drugs since start of the collaborative drug database (1st January 1995). The MaAsLin package was used to determine genera that caused the largest differences between groups [37]. In this analysis, the time category of antimicrobial drug use was linearly included as variable of interest, and age, sex, diabetes and kidney function were included as confounders. A *p*-value < 0.05 was considered statistically significant.

## Abbreviations

CKD-EPI: Chronic Kidney Disease Epidemiology Collaboration
DNA: Deoxyribonucleic acid
ERGO: Erasmus Rotterdam Gezondheid Onderzoek (Erasmus Rotterdam Health Research)
GP: General practitioner
OTU: Operational Taxonomic Unit
QIIME: Quantitative Insights Into Microbial Ecology
RNA: Ribonucleic acid
UTI: Urinary tract infection
